# Clinical outcome following best medical management in acute stroke with a proximal isolated occlusion of the anterior cerebral artery: an international multicentre study

**DOI:** 10.1093/esj/aakag014

**Published:** 2026-03-14

**Authors:** Candice Sabben, Frédérique Charbonneau, Michael Obadia, Davide Strambo, Elodie Ong, Mirjam R Heldner, Igor Sibon, Hilde Henon, Gioia Mione, Adrien Ter Schiphorst, Denis Sablot, Charlotte Rosso, Thomas Agasse-Lafont, David Weisenburger Lile, Jérémie Papassin, Solène Moulin, Aude Triquenot-Bagan, Loïc Legris, Nour Nehme, Valérie Wolff, Cécile Preterre, Roxana Poll, Yannick Béjot, Pierre Garnier, Guillaume Turc, Mikael Mazighi, Pierre Seners

**Affiliations:** Neurology Department, Rothschild Foundation Hospital, Paris, France; Neuroradiology Department, Rothschild Foundation Hospital, Paris, France; Neurology Department, Rothschild Foundation Hospital, Paris, France; Stroke Center, Neurology Service, Lausanne University Hospital and University of Lausanne, Lausanne, Switzerland; Stroke Department, Hospices Civils de Lyon, Lyon, France; Department of Neurology, University hospital and University of Bern, Bern, Switzerland; Stroke Unit, Bordeaux University Hospital, Bordeaux, France; Neurology Department, Stroke Center, University of Lille, Inserm U1171, CHU Lille, LilNCog - Lille Neuroscience & Cognition, Lille, France; Neurology Department, University Hospital of Nancy, Nancy, France; Neurology Department, CHRU Gui de Chauliac, Montpellier, France; Neurology Department, CH Perpignan, Perpignan, France; Urgences Cérébro-Vasculaires, DMU Neurosciences, Hôpital Pitié-Salpêtrière, AP-HP, Paris, France; STARE team, iCRIN, Institut du Cerveau (ICM), Sorbonne Université, Inserm U 1127, CNRS UMR 7225, GRC Nova, Paris 75013, France; Neurology Department, CHU Rennes, Rennes, France; Neurology Department, Foch Hospital, Suresnes, France; Neurology Department, CH Metropole Savoie, Chambery, France; Neurology Department, CHU Reims, Reims, France; Neurology Department, Rouen University Hospital, Rouen F-76000, France; Grenoble Institut Neurosciences, Inserm, U1216, Université Grenoble Alpes, CHU Grenoble Alpes, Grenoble 38000, France; Neurology Department, André Mignot Hospital, Versailles, France; Unité Neuro-Vasculaire, EA 3072, FMTS, Université de Strasbourg, Strasbourg, France; Neurology Department, CHU Nantes, Nantes, France; Neurology Department, Rene Dubois Hospital, Pontoise, France; Service de Neurologie, CHU Dijon Bourgogne, PEC2 UR7460, Université Bourgogne Europe, Dijon 21000, France; Stroke Unit, Neurology Department, CHU St Etienne, Saint-Priest-en-Jarez, France; FHU Neurovasc 2030, Paris, France; Neurology Department, GHU Paris Psychiatrie and Neurosciences, Paris, France; Institut de Psychiatrie et Neurosciences de Paris, Inserm U 1266, Université Paris Cité, Paris, France; FHU Neurovasc 2030, Paris, France; Interventional Neuroradiology Department, Rothschild Foundation Hospital, Paris, France; Université Paris Cité, Inserm 1144, Paris, France; Neurology Department, Lariboisière Hospital, APHP Nord, Paris, France; Institut Universitaire de France, Paris, France; Neurology Department, Rothschild Foundation Hospital, Paris, France; Institut de Psychiatrie et Neurosciences de Paris, Inserm U 1266, Université Paris Cité, Paris, France; Université Paris Cité, Inserm 1144, Paris, France

**Keywords:** acute stroke therapy, anterior cerebral artery, prognosis, stroke, thrombolysis

## Abstract

**Introduction:**

Acute ischaemic strokes (AIS) due to proximal anterior cerebral artery (ACA) occlusions are rare. Their clinical outcomes following medical management alone have been scarcely described.

**Patients and methods:**

We conducted a retrospective, multicentre, international study of consecutive AIS due to isolated proximal ACA occlusion (A1 or A2 segment) admitted within 6 h of symptom onset and treated with best medical management alone (ie, without endovascular therapy), across 23 centres in France and Switzerland. The primary outcome was poor functional outcome, defined as a mRS score > 2 at 3 months or failure to return to baseline mRS if the pre-stroke mRS was > 2. Associations between baseline clinical/radiological variables and outcome were evaluated in multivariable logistic regression analyses. Associations between outcome and key radiological follow-up variables such as recanalisation and haemorrhagic transformation were also analysed.

**Results:**

Ninety-five patients were included in the study: median age was 76 (IQR, 66–87), baseline NIHSS score was 10 (IQR, 5–15) and occlusion site was A1 in 8 (8%) and A2 in 87 (92%). Intravenous thrombolysis was administered in 76 (80%) cases. Poor functional outcome was observed in 47 (49%) patients. Among baseline variables, older age (adjusted odds ratio [aOR] per 5-year increase = 1.25; 95% CI, 1.10–1.55; *P* = .028) and higher NIHSS score (aOR = 1.20, 95% CI, 1.07–1.34; *P* < .001) were independently associated with poor outcome. Lack of recanalisation at 24 h was also independently associated with poor outcome (aOR = 14.5, 95% CI, 1.1–188.7, *P* = .04). Poor outcome was higher in patients with than in those without haemorrhagic transformation (73% vs 42%, *P* = .03) in univariable analysis, but not in multivariable analysis adjusting for age and NIHSS score (aOR = 2.3, 95% CI, 0.5–11.5, *P* = .32).

**Discussion and conclusion:**

Nearly half of AIS patients with isolated proximal ACA occlusion treated with medical management alone had poor 3-month functional outcomes. Older age, high NIHSS at admission and lack of recanalisation at 24 h were associated with poor outcome. These results underscore the need to investigate therapeutic strategies aimed at enhancing early arterial recanalisation to improve recovery in this population.

## Graphical Abstract

**Graphical Abstract:**
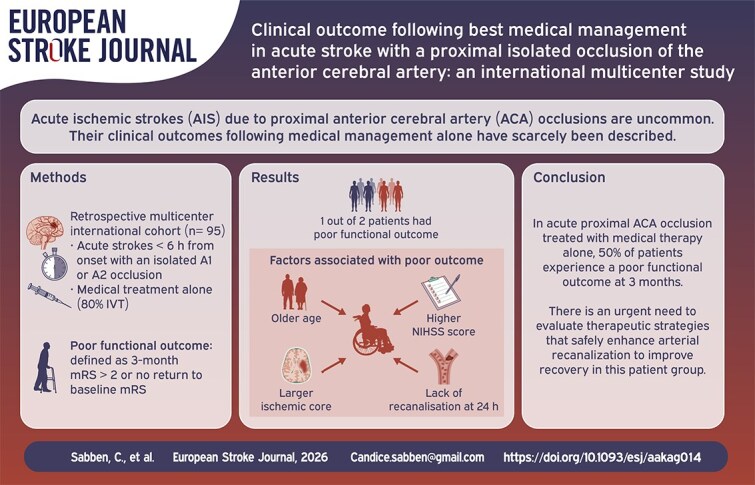

## Introduction

Acute ischaemic stroke (AIS) due to isolated occlusion of the anterior cerebral artery (ACA) is an uncommon clinical entity, accounting for 0.5%–3% of all ischaemic strokes.^1–3^ Anterior cerebral artery-related stroke may lead to neurologic deficits which substantially compromise patient’s autonomy and quality of life, encompassing motor deficits and a broad spectrum of neuropsychological impairments such as memory loss, frontal disinhibition and emotional lability.^3^ To date, there is a lack of robust observational studies describing the mid- or long-term functional outcome following acute isolated ACA occlusion, or deciphering the baseline clinical or radiological predictors of poor prognosis.^4^ Improving our understanding in this area is clinically relevant, as reliable early prognostication could guide communication with relatives about the patient’s prognosis during the acute phase and help identify patients’ subgroups for whom more aggressive therapies should be evaluated in future clinical trials to improve functional outcome.

Regarding acute revascularisation therapies, intravenous thrombolysis (IVT) is currently recommended for ACA strokes within the 4.5 h time window.^5^ While a few case series have suggested the feasibility and potential benefit of EVT in stroke with ACA occlusions,^6–8^ 2 recent randomised trials failed to demonstrate improved outcomes following EVT in AIS due to medium or distal vessel occlusions, yet ACA cases were underrepresented.^9^^,^^10^

In this study, we aimed to describe the 3-month functional outcome of AIS patients due to isolated proximal ACA occlusion admitted within the early time window and treated with medical therapy alone, using data from a large, international, multicentre cohort. In addition, we sought to identify the baseline clinical and radiological factors associated with poor functional outcome. Finally, we explored the association between poor outcome and key follow-up radiological variables, which may serve as potential therapeutic targets in future trials.

## Patients and methods

This analysis was reported according to the STROBE (Strengthening the Reporting of Observational Studies in Epidemiology) guidelines for observational studies.^11^

### Study cohort

This international multicentre retrospective study collected data from all consecutive acute stroke patients admitted to 23 stroke centres (France, *n* = 21; Switzerland, *n* = 2) between February 2003 and January 2022 (inclusion dates varied across centres), who met the following criteria: (1) baseline non-invasive brain and arterial imaging (CT with CT-angiography or MRI with MR-angiography) performed within 6 h of symptom onset and showing an isolated proximal occlusion of the first (A1) or second (A2) segment of the ACA, (2) treated with medical management alone (including IVT if indicated). Patients receiving EVT or with missing 3-month mRS scores were excluded. Given the absence of guideline-based recommendations for EVT in isolated ACA occlusions throughout the study period, EVT was considered only on a case-by-case basis in some centres. Three participating centres were unable to retrieve data from patients presenting ACA stroke receiving neither IVT nor EVT.

The study was approved by the local ethics committee of the Rothschild Foundation Hospital (IRB-00012801). Each patient was informed of her/his participation in the study and was offered the possibility to withdraw, if required by the local legislation. The data supporting the study findings are available upon reasonable request.

### Clinical and radiological variables

Clinical variables routinely recorded in the acute stroke setting were recorded (age, sex, pre-stroke mRS, vascular risk factors, previous vascular events, previous antithrombotic treatments, NIHSS score on admission, time between symptom onset and admission imaging, 3-month mRS and aetiology of the stroke according to the Trial of ORG 10172 in Acute Stroke Treatment [TOAST] classification).

All included patients underwent either non-contrast CT with CT-angiography or MRI with MR-angiography on admission, and follow-up MRI or CT within ~ 24 h following admission. To ensure homogeneity in radiological evaluation, 1 neuroradiologist with 15 years of experience (F.C.) reviewed all baseline and follow-up imaging, blinded to clinical outcomes.

The following variables were collected: First, the site of ACA occlusion on CT-angiography or MR-angiography. The A1 segment was defined as the horizontal pre-communicating A1 segment which courses over the optic nerve and chiasm, and the A2 segment as the vertical post-communicating which enters into the interhemispheric fissure, and terminates at the junction of the rostrum and genu of the corpus callosum.^12^^,^^13^ Second, the infarct core volume on DWI or CT-perfusion, whenever available. On DWI, infarct volume was manually outlined based on DWI signal intensity encompassing the entire area of bright DWI signal intensity. On CT-perfusion, infarct volume was automatically measured using RAPID software using the relative cerebral blood flow < 30% of normal brain. Artefacts were removed manually. Third, on 24-h imaging, evidence of haemorrhagic transformation (HT) according to the European Cooperative Acute Stroke Study (ECASS) II classification.^14^ Last, evidence of partial or complete recanalisation on 24-h arterial imaging, when available, defined as a score 2b or 3 on the revised Arterial Occlusive Lesion scale.^15^

### Study outcome

The primary outcome was 3-month poor functional outcome, defined as mRS > 2 or no return to pre-stroke mRS for patients with pre-stroke mRS > 2.^16^

### Statistical analysis

Univariable relationships between baseline clinical or radiological characteristics and poor 3-month functional outcome were assessed using the Mann–Whitney *U* test for continuous variables, and the chi-square test or Fisher’s exact test for categorical variables, as appropriate. To adjust for potential confounders, multivariable binary logistic regression analysis was performed with poor 3-month outcome as the dependent variable. A stepwise variable selection process was performed, whereby variables that were significant at *P* < .20 in the univariable analysis were entered into the model and were retained only if they remained associated at *P* < .05 with the dependent variable in the multivariable model. Because baseline NIHSS score and infarct core volume are strongly correlated, they were not entered simultaneously into the multivariable model to avoid collinearity. Also, the relationship between 2 key early follow-up radiological variables (namely, 24-h recanalisation and any HT) and poor 3-month outcome were studied in univariable analysis then multivariable binary logistic regression analysis, adjusting for age and baseline NIHSS score. All statistical tests were 2-tailed, and the threshold for statistical significance was set to *P* < .05. Statistical analyses were conducted using SPSS 29.0 (IBM, Armonk, NY).

## Results

### Study population

**Figure 1:**
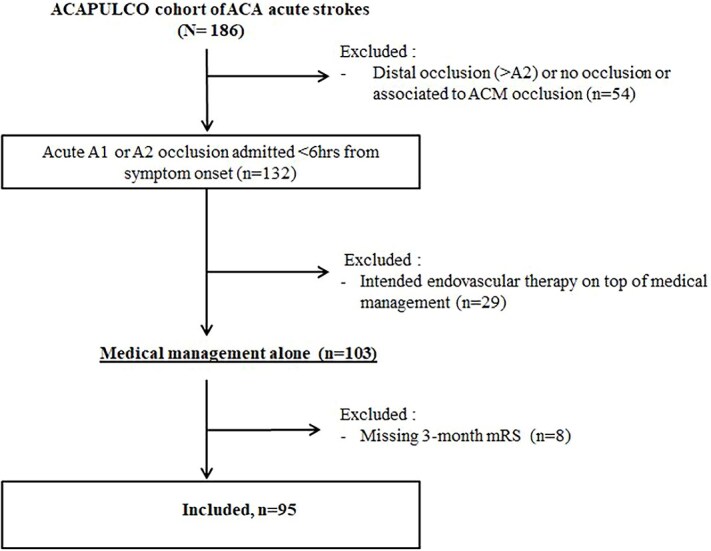
Flow chart of the study.

Of the 132 patients with an isolated A1 or A2 occlusion, 37 were excluded (29 received EVT, and 8 had missing 3-month mRS), leaving 95 for the analysis (see the flowchart in Figure 1). Compared with included patients, those excluded were more often treated after 2015 and less likely to receive IVT; otherwise, baseline characteristics were similar between groups (Table S1).

Median age was 76 years (IQR, 66–87), 37 (39%) were female and median baseline NIHSS score was 10 (IQR, 5–15). Women were significantly older than men in our cohort, with a median age of 86 years (IQR, 75–90) compared with 73 years (IQR, 63–80), respectively. Baseline imaging was performed at a median delay of 126 min (IQR, 98–161) following symptom onset. Occlusion site was A1 in 8 (8%) and A2 in 87 (92%) patients, respectively. Median baseline infarct volume, available in 86 (91%) patients, was 12 mL (IQR, 0–30). Intravenous thrombolysis was administered in 76 patients (80%). Stroke aetiology was cardio-embolic in 47 patients (50%), atherosclerotic in 8 (8%) and undetermined in 37 (39%).

Poor functional outcome (mRS > 2 or failure to return to baseline in patients with pre-stroke mRS > 2) was observed in 47 patients (49%). At 3 months, 12 (13%) patients had died.

### Baseline factors associated with poor functional outcome

In univariable analysis, several differences in baseline characteristics were observed between patients with and without poor 3-month functional outcome (Table 1). Compared to patients with a good 3-month outcome, those with a poor outcome were more often female, older, more frequently on anticoagulant therapy at admission, and had higher pre-stroke mRS and baseline NIHSS scores. On baseline imaging, they exhibited larger infarct core volumes and more often had left-sided occlusions. The rate of IVT, the site of occlusion (A1 vs A2) and delays from symptom onset to imaging or to thrombolysis did not significantly differ between the 2 groups.

**Table 1:** Univariable comparison of baseline characteristics according to 3-month functional outcome.

	Total (*n* = 95)	Poor outcome *(3-month mRS 3–6 and no return to baseline mRS)* (*n* = 47)	Good outcome *3-month mRS 0–2 or return to baseline mRS)* (*n* = 48)	*P*
**Female**	37 (39%)	24 (51%)	13 (27%)	.02
**Age, years (IQR)**	76 (66–87)	86 (74–90)	72 (57–81)	<.01
**Pre-stroke mRS**	0 (0-2)	1 (0–3)	0 (0–1)	.01
**Hypertension**	78 (82%)	39 (83%)	39 (81%)	.83
**Diabetes**	23 (24%)	11 (23%)	12 (25%)	.86
**Active smoking**	9 (10%)	2 (4%)	7 (15%)	.16
**Previous atrial fibrillation**	25 (26%)	14 (30%)	11 (23%)	.45
**Previous stroke**	18 (19%)	11 (23%)	7 (15%)	.27
**Previous coronary disease**	17(18%)	8 (17%)	9 (19%)	.83
**Pre-stroke antiplatelet**	39 (41%)	22 (47%)	17 (35%)	.26
**Pre-stroke anticoagulant**	8 (8%)	7 (15%)	1 (2%)	.03
**NIHSS score at admission**	10 (5–15)	15 (10–18)	7 (3–11)	<.01
**SBP at admission**	150 (132–160)	155 (134–161)	145 (128–160)	.25
**DBP at admission**	80 (69–97)	82 (70–98)	79 (67–91)	.49
**Onset-to-imaging, min**	126 (98–161)	129 (81–169)	109 (81–144)	.15
**Admission in a comprehensive stroke centre**	85 (90%)	39 (83%)	46 (96%)	.05
**After 2015**	64 (67%)	35 (75%)	29 (60%)	.14
**Occlusion site**				>.99
** A1**	8 (8%)	4 (8%)	4 (8%)	
** A2**	87 (92%)	43 (92%)	44 (92%)	
**Occlusion side**				.04
** Left**	57 (60%)	34 (72%)	23 (48%)	
** Right**	38 (40%)	13 (28%)	25 (52%)	
**Infarct volume,^a^ mL**	12 (0–30)	19 (7–39)	5 (0–18)	.01
**Intravenous thrombolysis**	76 (80%)	38 (81%)	38 (79%)	.84
**Onset-to-needle time, min**	163 (131–194) *n* = 76	170 (135–197) *n* = 38	150 (123–189) *n* = 38	.18
**Cardio-embolic aetiology**	47 (50%)	25 (53%)	22 (46%)	.47

^a^Infarct volume was available in 86 (91%) patients (42 [88%] with good outcome and 44 [94%] with poor outcome).

Abbreviations: A1 = first segment of ACA; A2 = second segment of ACA; DBP = diastolic blood pressure; SBP = systolic blood pressure.

In multivariable analysis, only age (adjusted odds ratio [aOR] per 5-year increase = 1.25; 95% CI, 1.10–1.55; *P* = .028) and baseline NIHSS score (aOR per 1-point increase = 1.20; 95% CI, 1.07–1.34; *P* < .001) were independently associated with poor 3-month outcome. In an alternative model in which baseline NIHSS score was replaced by infarct core volume, age (aOR per 5-year increase, 1.38; 95% CI, 1.13–1.68; *P* = .001), infarct core volume (aOR per 5-mL increase, 1.19; 95% CI, 1.03–1.38; *P* = .017) and left-sided occlusion (aOR, 3.50; 95%CI, 1.23–9.98; *P* = .019) were independently associated with poor 3-month outcome.

### Follow-up radiological variables associated with poor functional outcome

#### 24-h recanalisation

**Figure 2:**
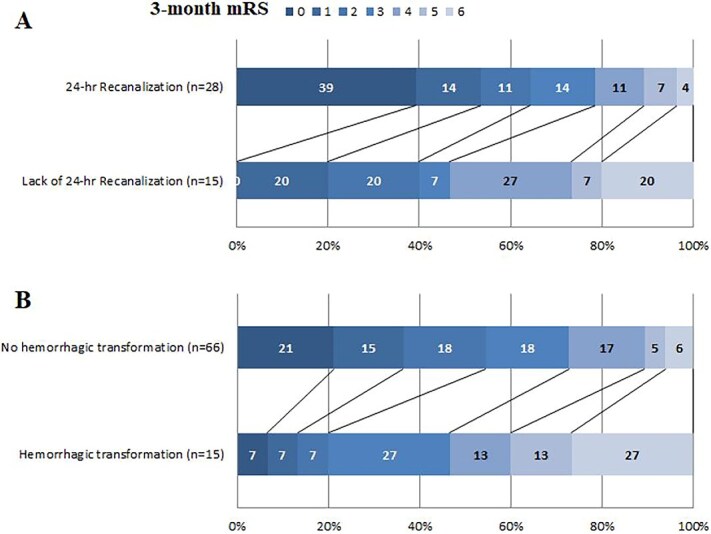
Relationship between 3-month functional outcome and 24-h recanalisation **(2A)** or haemorrhagic transformation **(2B)**.

Follow-up arterial imaging at 24-h was available in 43 (45%) patients. Patients with recanalisation assessment had similar baseline characteristics as compared to those without (Table S2). Arterial recanalisation was observed in 28/43 (65%) patients. The 3-month mRS distribution in patients with and without recanalisation is presented in Figure 2A. In univariable analysis, poor outcome was numerically higher in patients without (7/15, 47%) than in those with (9/28, 32%) recanalisation (*P* = .18). Following adjustment for age and baseline NIHSS, the lack of recanalisation was independently associated with poor outcome (aOR = 14.5; 95% CI, 1.1–188.7; *P* = .04).

#### Haemorrhagic transformation

Follow-up brain imaging at 24 h was available in 81 (85%) patients. Patients with follow-up brain imaging had similar baseline characteristics as compared to those without, except for more frequent IVT use (Table S3). Any HT was observed in 15/81 (19%) patients: 7 haemorrhagic infarction type 1, 5 haemorrhagic infarction type 2 and 3 parenchymal haematoma type 1. No parenchymal haematoma type 2 occurred. The 3-month mRS distribution in patients with and without HT is presented in Figure 2B. Poor outcome was higher in patients with than in those without HT (11/15 [73%] vs 28/66 [42%], *P* = .03) in univariable analysis, but not in multivariable analysis adjusting for age and NIHSS score (aOR = 2.3; 95% CI, 0.5–11.5; *P* = .32).

## Discussion

This study presents, to our knowledge, the largest multicentre cohort to date assessing predictors of poor 3-month functional outcome in patients with AIS due to an isolated proximal ACA occlusion treated with medical management alone. Nearly half of the patients had a poor 3-month outcome, and 13% died, highlighting the clinical severity of ACA strokes and the urgent need to optimise care strategies in this population.

A smaller Korean cohort of 47 patients with ACA infarcts—though not limited to those admitted in the hyperacute setting—, recently reported 85% of favourable functional outcome at 3 months.^4^ However, the cohort included both patients with and without proximal ACA occlusion, thereby capturing milder cases. Notably, the authors observed that a proximal ACA occlusion was associated with a 17-fold increase in the odds of substantial disability. Interestingly, despite favourable 3-month mRS scores, nearly half of the patients experienced a change in dwelling status and job loss, underscoring persistent disability. These findings highlight the limitations of the mRS in capturing cognitive and behavioural sequelae commonly associated with ACA strokes. Complementary tools such as the Montreal Cognitive Assessment, Frontal Assessment Battery, or quality-of-life scales may offer a more comprehensive evaluation of recovery in this population in this context.^17^

In our cohort, older age, higher baseline NIHSS and larger core volume remained independent predictors of outcome, consistent with robust data from middle cerebral artery or posterior circulation stroke populations^18^^,^^19^ and reaffirming their prognostic value in ACA strokes. Left-sided occlusion was independently associated with poor outcome in the multivariable model including infarct core volume, but not in the model including NIHSS score. This is likely because the baseline NIHSS score captures both ischaemic severity and functional topography, with left-hemispheric strokes being more clinically eloquent—particularly due to language deficits—than right-sided strokes. Although female sex was associated with poor outcome in univariable analysis, this association did not persist after adjustment for age, suggesting that the observed sex difference was largely driven by older age at presentation rather than by sex itself.

We also identified an association between the absence of 24-h recanalisation and poor 3-month outcome. Although this finding should be interpreted with caution because of the limited sample size—only 45% had follow-up vascular imaging—and the resulting wide 95% CIs, it is consistent with well-known observations in other occlusion locations.^20^ This finding suggests that interventions enhancing recanalisation rates in this population may help reduce disability. Although a few case series have supported the feasibility and potential benefit of EVT,^6–8^ randomised controlled data are scarce. Two recent trials failed to demonstrate improved outcomes following EVT in AIS due to medium or distal vessel occlusions^9^^,^^10^; however, ACA cases were underrepresented (31 A1–A3 occlusions in DISTAL, and 24 A2 and A3 occlusions in ESCAPE-MeVO). A subgroup analysis on ACA occlusions was reported only in the DISTAL trial, where the point estimate did not favour EVT (OR, 0.43; 95% CI, 0.10–1.83). Regarding IVT, its use was not associated with improved outcomes in our cohort, which may reflect limited statistical power. There is a pressing need for research into intravenous and endovascular strategies that can optimise recanalisation without compromising safety.

Our study has limitations. First, its retrospective nature with unblinded mRS assessment limits conclusions. A causal relationship between recanalisation or HT and poor functional outcome cannot be established. Second, despite the multicentre design, our sample size was moderate due to the rarity of this type of stroke, and small but potentially clinically relevant associations may therefore have been missed. In addition, the use of stepwise regression in a limited sample may have led to data-driven and unstable variable selection with limited robustness and reproducibility; therefore, confirmation in larger, independent datasets is required. Third, the inclusion period spanned nearly 2 decades, during which stroke management evolved substantially—particularly with respect to imaging protocols, the use of IVT and the availability of EVT—which may have influenced our results. Moreover, patients were treated in 23 different sites, which may introduce variation in care practices. Fourth, the retrospective design precluded the collection of detailed and standardised data on all components of best medical management beyond IVT, including antithrombotic strategies, blood pressure management and early rehabilitation. Last, 3 out of 23 centres were unable to retrieve data for patients who received neither IVT nor EVT, potentially leading to an underrepresentation of patients who did not undergo either therapy.

## Conclusion

In this multicentre cohort of AIS patients with isolated proximal ACA occlusion admitted within the early time window and managed medically, a poor 3-month functional outcome was observed in 1 out of 2 patients. Older age and higher baseline NIHSS score were independent predictors of unfavourable outcome. In addition, the lack of arterial recanalisation was associated with poor prognosis. These findings highlight the need to evaluate therapeutic strategies aimed at promoting arterial recanalisation to improve outcomes in this patient population.

## Supplementary Material

ESJ_Supplemental_Materials_ACA_rvf_(3)_aakag014
