# Supplementary material for: Clinical outcome following best medical management in acute stroke with a proximal isolated occlusion of the anterior cerebral artery: an international multicentre study
Source: Eur Stroke J. 2026 Mar 14;11(3):aakag014. doi: 10.1093/esj/aakag014 (PMC12988719; doi:10.1093/esj/aakag014)
Supplement: ESJ_Supplemental_Materials_ACA_rvf_(3)_aakag014 [file esj_supplemental_materials_aca_rvf_(3)_aakag014.docx]

**Supplemental Results**

**Supplemental Table 1. Comparison of included *vs.* excluded patients**

|  | **Included**  **n=95** | **Excluded**  **n=37** | ***P* value** |
| --- | --- | --- | --- |
| Age, years | 76 (66-87) | 81 (71-86) | 0.438 |
| Female | 37 (39%) | 19 (51%) | 0.240 |
| NIHSS score | 10 (5-15) | 9 (6-15) | 0.588 |
| Baseline Imaging is MRI | 58 (61%) | 26 (70%) | 0.421 |
| Onset to imaging time, hrs. | 126 (98-161) | 129 (86-184) | 0.262 |
| Occlusion site |  |  | 0.214 |
| A1 | 8 (6%) | 7 (19%) |  |
| A2 | 87 (92%) | 30 (81%) |  |
| Intravenous thrombolysis | 76 (80%) | 19 (51%) | 0.002 |
| After 2015 | 64 (67%) | 36 (97%) | <0.001 |

*Among the 37 excluded patients, 29 were excluded because they received endovascular therapy and 8 due to missing 3-month mRS data.

**Supplemental Table 2. Comparison of patients with and without 24-hr recanalization assessment**

|  | **24-hr recanalization assessment**  **n=43** | **Lack of 24-hr recanalization assessment**  **n=52** | ***P* value** |
| --- | --- | --- | --- |
| Age, years | 73 (67-88) | 79 (67-88) | 0.120 |
| Female | 17 (39.5%) | 20 (38.5%) | 0.915 |
| NIHSS score | 10 (4-15) | 10 (8-17) | 0.88 |
| Baseline imaging is MRI | 23 (53.5%) | 35 (67.3%) | 0.169 |
| Onset to imaging time, hrs. | 128 (81-160) | 121 (86-152) | 0.927 |
| Occlusion site |  |  | 0.779 |
| A1 | 4 (9.3%) | 4 (7.7%) |  |
| A2 | 39 (90.7%) | 48 (92.3%) |  |
| Intravenous thrombolysis | 35 (81.4%) | 41 (78.8%) | 0.757 |

**Supplemental Table 3. Comparison of patients with and without 24-hr follow-up brain imaging**

|  | **24-hr follow-up brain imaging**  **n=81** | **Lack of 24-hr follow-up brain imaging**  **n=14** | ***P* value** |
| --- | --- | --- | --- |
| Age, years | 75 (65-86) | 83(75-90) | 0.136 |
| Female | 30 (37%) | 7 (50%) | 0.358 |
| NIHSS score | 10 (5-15) | 12 (7-19) | 0.943 |
| Baseline imaging is MRI | 51 (63%) | 7 (50%) | 0.358 |
| Onset to imaging time, min | 125 (90-160) | 96 (0.2-139) | 0.248 |
| Occlusion site |  |  | 0.392 |
| A1 | 6 (7.4%) | 2 (14.3%) |  |
| A2 | 75 (92.6%) | 12 (85.7%) |  |
| Intravenous thrombolysis | 70 (86.4%) | 6 (42.9%) | <0.001 |
